# Vasoplegia Following Complex Spine Surgery: Incidence and Risk

**DOI:** 10.1177/21925682221105823

**Published:** 2022-05-29

**Authors:** Basem Ishak, Juan N. Pulido, Alexander von Glinski, Darius Ansari, Rod J. Oskouian, Jens R. Chapman

**Affiliations:** 1 Department of Neurosurgery, Heidelberg University Hospital, Heidelberg, Germany; 2 Swedish Neuroscience Institute, Swedish Medical Center, Seattle, WA, USA; 3 Swedish Medical Center, Cardiothoracic Anesthesiology and Critical Care Medicine, Seattle, WA, USA; 4 BG University Hospital Bergmannsheil, Ruhr University, Bochum, Germany

**Keywords:** vasoplegia, vasodilatory shock, hypotension, thoraco-lumbar fusion, complications, vasopressors, ICU management

## Abstract

**Study design:**

Retrospective cohort study

**Objectives:**

Vasoplegia is a life-threatening form of distributive or vasodilatory shock that is characterized by reduced systemic vascular resistance with resultant hypotension and normal to elevated cardiac output affecting morbidity and mortality. Vasoplegia in the context of Spine Surgery has not been described previously. The purpose of this case series is to determine incidence, risk factors, complications and postoperative outcome in patients with vasoplegia after complex multi-level thoraco-lumbar spine surgery.

**Methods:**

A retrospective review of the electronic medical records at our institution was conducted between January 2014 and June 2018. All patients undergoing multi-level spine surgery (>6 levels) were screened for intraoperative hypotension. Patient demographics, surgical characteristics, neurological status, blood loss, risk factors, medical treatment, complications, hospital course and mortality were collected. All patients included in this study had a minimum follow-up period of 3 months.

**Results:**

Out of 8521 surgically treated patients, 994 patients with multi-level thoraco-lumbar spine surgery were identified. A total of 41 patients had intraoperative hypotensive events. Of those, 5 patients with vasoplegia could be identified after elimination of all other potential contributing factors. Vasoplegia did not influence the neurological outcome. One major and three minor complications occurred. All patients showed full recovery. The risk factors identified for vasoplegia include prolonged surgery with osteotomies.

**Conclusions:**

Vasoplegia is a rare condition with an incidence of .6%. Patients experiencing vasoplegia did not appear to experience worse surgical outcomes. The use of special intraoperative hemodynamic monitoring should be considered in selected cases.

## Introduction

We are introducing the concept of vasoplegia and its potential manifestation in major spine surgery in this retrospective study. Vasoplegia is a form of distributive or vasodilatory shock that has most commonly been described in the perioperative setting of patients undergoing Cardio-thoracic Surgery.^1-3^ The incidence of vasoplegia after Cardiac Surgery has been reported in up to 25% of patients, accompanied by a high complication, morbidity and mortality rate.^3-6^

Compared to other forms of perioperative hypotension (eg cardiogenic, hypovolemic and obstructive shock), vasoplegia occurs after adequate fluid resuscitation and in the presence of normal or high cardiac output, with reduced response to conventional vasopressors such as norepinephrine that ultimately contributes to the shock state.^1,2^ The hallmark of vasoplegia is the failure of the vasculature to maintain an adequate systemic vascular resistance (SVR).^5,7^

Although the exact mechanism is unknown, it is suggested that vasoplegia is associated with a systemic inflammatory response, resulting in endothelial dysfunction and enhanced nitric oxide (NO) synthesis, ultimately increasing the production of cyclic guanosine monophosphate (cGMP). Elevated levels of NO and cGMP lead to inappropriate vasodilatation refractory to commonly used vasopressors.^8,9^

Although its incidence is rarely reported, risk factors for vasoplegia following major non-cardiac surgery include prolonged surgery duration and significant blood transfusion requirements.^2^ Complex Spine Surgery—including procedures for deformity, neoplastic, and infectious diseases requiring spinal column reconstruction—harbors known risk factors for the development of vasoplegia. There are currently no available studies that describe vasoplegia in the context of Spine Surgery.

Despite this paucity within the literature, there is value in studying the incidence, risk factors, and management strategies of vasoplegia so that Spine Surgeon and perioperative team members, can plan accordingly and consider enhanced hemodynamic monitoring and implement potential treatment responses. Hypotension and vasoplegia events inherently carry significant relevance for complex spine surgery due to the vulnerability of spinal neural elements to diminished perfusion and potential subsequent neurologic injury. Further understanding of the pathophysiology, prevention strategies, and avenues for ongoing management surrounding vasoplegia could potentially prevent avoidable post-operative neurologic injury in complex spine patients and decrease duration of intensive care as well as decrease other potentially serious complications.

The purpose of this retrospective study is to determine the incidence of vasoplegia in complex Spine Surgery patients in a relatively controlled population, identify associated risk factors, morbidity, mortality and adverse neurologic outcomes in patients with this diagnosis. Furthermore, we hypothesize that vasoplegia occurs in patients undergoing multilevel thoraco-lumbar fusion and is associated with a high morbidity rate.

## Methods

After institutional review board approval (ID: STUDY2018000464) a retrospective review of the electronic medical records of 8521 patients who underwent surgical treatment for spine pathologies at our institution from January 2014 to June 2018 was performed. All patients who underwent multi-level spine surgery (>6 motion levels) were screened for intraoperative hypotension, requirement of vasopressors for at least 24 hours and mean arterial pressure gradients (MAP) < 60 mmHg for > than 2 consecutive hours. Selection was performed by a multispecialty group of experts (BI, JP, AVG). Vasoplegia was defined as hypotension requiring a vasopressor and not associated with hypovolemia (CVP >10 mmHg). Furthermore, vasoplegia was categorized into a) mild: MAP 50-60 mmHg and one vasopressor, b) moderate: MAP 50-60 mmHg and two vasopressors and c) severe: MAP <50 mmHg and two or more vasopressors.

To avoid additional confounding variables patients younger than 18 years and patients treated for acute trauma, tumor, or infections were excluded from the study. Furthermore, we excluded any patients with a known history of systolic heart failure (left ventricular ejection fraction <40% or right ventricular dysfunction), and those requiring postoperative vasopressors for reasons other than vasoplegia. Inclusion and exclusion criteria are depicted in Table 1. Informed consent was not required for this type of retrospective study.

**Table 1.** Inclusion/Exclusion criteria for identifying vasoplegia.

Inclusion Criteria	Exclusion Criteria
**Age > 18 years**	Trauma, tumor, infection
**Multi-level thoraco-lumbar fusion (>6 levels)**	Rheumatoid arthritis
**MAP < 60 mmHg for > than 2 consecutive hours**	Cervical spine surgery
**Continuous use of vasopressors in ICU (>24h)**	History of diminished cardiac output
	Previously known hypotension
	Pevious spine surgery

To identify possible contributory patient characteristics, we collected patient demographics, body mass index (BMI), American Society of Anesthesiologists (ASA) score, relevant medical risk factors, comorbidities, preoperative medications, surgical treatment, duration of surgery, intraoperative estimated blood loss, intra- and post-operative blood transfusions, management of intraoperative fluids, complications, mortality rate, length of hospital stay, and discharge disposition as documented in the patients’ electronic medical records.

Neurological assessment was based on the Motor Score which was recorded on admission, at discharge, and at 90 days follow-up.

Further attention was paid to the vasopressors administered in the intensive care unit (ICU) and responses of any secondary consultant Cardiology services including repeat 12 lead ECG and echocardiography.

### Endpoints

Primary endpoint was to identify the incidence and risk factors of vasoplegia during elective complex spinal surgical procedures. Neurological outcome, morbidity and mortality rate were considered as secondary endpoints at 3-months follow-up.

### Statistical Analysis

Means and standard deviations were computed for descriptive analysis. SPSS 22 (SPSS Munich, Germany) was used for all statistical analyses. A paired t-test for two related samples was used to determine a statistical difference across the time in the same group. A *P*-value less than .05 was determined a priori to represent statistical significance. All data were analyzed by an independent external statistician.

## Results

Out of 8521 patients who received spine fusion surgery at our tertiary/quaternary referral center, we identified 994 patients with multi-level thoraco-lumbar spine surgery (>6 levels) during the study period. Of this cohort, 41 patients (4.1%) experienced unexpected intraoperative hypotension treated with vasopressors. A detailed description of underlying causes and mechanisms of hypotension is provided in Table 2. Vasoplegia occurred in 5 patients with an incidence of .6% in multi-level thoraco-lumbar fusion surgery after excluding all patients with potentially confounding covariable conditions.

**Table 2.** Underlying causes and mechanisms of intraoperative hypotension in patients with complex multi-level spine surgery (n=994).

Etiology	Frequency (%)	n=41
Hypovolemic shock **(hemorrhagic)**	15 (15%)	
Obstructive shock **(pulmonary embolism)**	2 (2%)	
Cardiogenic shock	4 (4%)	
Distributive shock **(septic)** **(neurogenic)** **(vasoplegic)**	13 (13%) 2 (2%) 5 (5%)	

### Patient Characteristics

Patients with vasoplegia showed no significant gender predilection (males 3/5), the average age was 67 ± 4.2 years (range 60-73 years) (Table 3). BMI ranged from 26-36 kg/m^2^ with a mean BMI of 30.7± 4.1 kg/m^2^. Preoperative narcotic-relevant ASA scoring revealed 1 patient (20%) with an ASA 3 score and 4 patients (80%) with an ASA 2 (Table 3).

**Table 3.** Baseline characteristics.

Patient Number	Age	Sex	BMI [kg/m2)	Diagnosis	Surgical Therapy	Duration of Surgery [min)	Estimated Blood Loss [Ml)	Blood Transfusion [Units)	Intaroperative i.v. Fluids [Ml)	
1	69	f	26.8	neuromuscular_kyphoscoliosis	T3-ilium	610	1500	4	9000	
2	73	m	29.1	Thoracic hyperkyphosis	T2-L1 (abandoned)	295	1000	6	500	
3	66	m	26.7	Thoraco-lumbar kyphoscoliosis	T8-pelvis	374	1500	2	7000	
4	60	f	36.5	Thoraco-lumbar kyphoscoliosis	T10-pelvis	364	800	1	5500	
5	67	m	34.6	Thoraco-lumbar kyphoscoliosis	T9-pelvis	481	1000	2	3500	

BMI= Body Mass Index; ASA= American Society of Anestiology; EBL= Estimated blood loss.

### Risk Factors

All 5 patients had an established history of well-controlled hypertension managed with either one or two antihypertensive agents (Table 3). Of these, 2 (40%) patients were smokers, and one (20%) had a history of diabetes. Chronic renal insufficiency and chronic obstructive pulmonary disease were present in one patient each. None of the patients had a history of prior cardiovascular disease.

### Surgical Treatment

All five patients received multi-level thoraco-lumbo-pelvic fusion surgery with either three-column or multi-level posterior column osteotomies for deformity correction. In one patient with thoracic kyphoscoliosis, surgery was abandoned due to hemodynamic instability which could not be controlled adequately with intravenous vasopressors in addition to fluid management. Intraoperative monitoring was used in all patients during deformity correction. A temporary signal loss occurred in two patients and significant deterioration of signals (>50%) were seen in three patients during correction maneuvers. After immediate release of correction forces, the signals showed full recovery at the end of surgery in all cases. The intra-operative blood loss ranged from 800 cc to 1500 cc with a mean blood loss of 1160 cc. All patients received intraoperative blood transfusions. Surgical time was 424.8 ± 110.1 min (range 295-610 min). Intraoperative complications occurred in one patient with cerebrospinal fluid leak, which was addressed intraoperatively without further sequelae.

### Postoperative Course

Postoperatively, all patients identified with vasoplegia had been placed in an intensive care unit. Vasopressors were administered for at least 24 hours. A single vasopressor (phenylephrine) was administered in two patients (*mild vasoplegia*). One patient (phenylephrine, norepinephrine) required two vasopressors (*moderate vasoplegia*). Three vasopressors (phenylephrine, vasopressin, norepinephrine) were administered in two patients (*severe vasoplegia*). None of these patients showed any abnormality on ECG or echocardiogram. After careful multisystems review no other reasons for this distributive shock could be identified. Mean ICU stay was 4.8± 3.1 days (range 2-9 days). Mean hospital stay was 11± 4.9 days (range 6-20) days.

### Clinical Outcome and Postoperative Complications

The mean preoperative motor score was 96 ± 4.9, which did not deteriorate postoperatively. At 3-months follow-up the mean motor score was 98 ± 4, which showed a slight improvement but without any statistical difference across the time (P>.05). Despite changes in intraoperative neurophysiologic monitoring, no substantive neurological deficits were observed in any patients suffering from vasoplegia.

One patient developed a major complication in form of a pulmonary embolism days after the shock was resolved, which was treated with low molecular weight heparin anti-thrombotic therapy. Minor complications occurred in three patients including urinary retention and encephalopathy. One patient experienced no complication. No in-hospital mortality occurred, and all patients recovered fully at the 90-day cut-off point for this study. Three patients were discharged to a postoperative rehabilitation unit, one to a skilled nursing facility, and one to home.

## Discussion

Vasoplegia is a pathologic state characterized by hypotension (MAP <60 mmHg) due to inappropriate reduction in systemic vascular resistance in the presence of normal to elevated cardiac output. It is best described in the cardiac surgery population due to a high release of inflammatory mediators as a result of direct patient contact with the bypass machine. While this is encountered relatively frequently in patients undergoing cardiopulmonary bypass surgery (CPB), with an incidence ranging from 5-25%,^5^ vasoplegia in the context of Spine Surgery has not yet been formally described. To this end, we identified patients undergoing elective multi-level reconstructive thoracolumbar spine fusion surgery at our institution and found the overall incidence of vasoplegia requiring some form of prolonged vasopressor administration to be .6%. The pathophysiologic mechanism central to the development of vasoplegia—lack of appropriate response to vasopressors—is commonly encountered in the setting of shock, acidemia, and in individuals who have received cardiopulmonary bypass surgery.^10^ In addition to these pathophysiologic mechanisms, autonomic nervous system dysregulation has to be considered in patients undergoing complex spine surgery. In this context, spinal cord injury, especially at the cervical and high thoracic levels, can also result in vasoplegia secondary to damage to the sympathetic nervous system.^11^ To get a better understanding of this complex mechanism, it is important to look at it from an anatomical point of view: The preganglionic sympathetic neurons are located within the lateral horn into the intermediolateral nuclei of T1-L2 cord segments.^12^ They exit the spinal cord via the ventral root, and synapse with postganglionic neurons within the paravertebral sympathetic chain. Postganglionic sympathetic fibers outflow through peripheral nerves to various sites, including the vessels and heart. Along with the well-known motor and sensory deficits, injury to these structures can be expected to result in corresponding autonomic disturbances.^13,14^ The autonomic nervous system, via its sympathetic and parasympathetic divisions, regulates circulatory function such as blood pressure and pulse.^15^ The sympathetic division is also responsible for increasing heart rate and peripheral vascular resistance through vasoconstriction. Because activating impulses are transmitted from the supraspinal centers to the preganglionic sympathetic neurons, interruption of this descending pathway may result in insufficiency of the sympathetic chain.^16^

In the presence of unopposed parasympathetic output from the vagal nerve, the relative sympathetic hypoactivity leads to several physiologic changes. As seen in patients with complete cervical motor SCI (ie ASIA A and B), manifestations of sympathetic dysfunction (and in severe cases, neurogenic shock) include bradycardia, arterial hypotension, and requirement of vasopressors in 35%. In thoracolumbar SCI bradycardia is less frequent and has been encountered in 13-35% cases.^17^

Although no patients in the present study encountered intraoperative complications, patients did receive multilevel thoraco-lumbo-pelvic fusion surgery with aggressive kyphoscoliotic deformity correction around the thoracolumbar junction. Disruption of the paravertebral sympathetic chain and/or accompanying manipulation of the spinal cord and cauda equina may therefore provide a direct neurologic cause for vasoplegia through temporary sympathetic dysfunction.^18,19^

Pharmacologic agents may also induce vasoplegic effects such as certain anesthetic agents, in example Propofol (N01AX10) or volatile anesthetics. These agents are known to negatively affect systemic vascular resistance, cardiac output and HR, and may have to be adjusted if vasoplegia is identified.^20^ However, these are all ubiquitous agents used in all patients included in the study at standard doses.

As to management of unexplained intraoperative hypotension by modifying intraoperative anesthetic agents, other options to treat suspected vasoplegia in the postoperative spine patients consist of rebalancing of catecholaminergic and non-catecholaminergic medications. Each of our patient was administered at least one vasopressor (Phenylephrine) for at least 24 hours, with monitoring for subsequent improvement in vital parameters; those failing to respond were escalated to two (Phenylephrine and Norepinephrine) or three (Phenylephrine, Norepinephrine, Vasopressin) agents. Vasopressin is formally approved by the United States Food and Drug Administration (FDA) for treatment of vasoplegia and should be considered as a second line agent (after initiation of Norepinephrine).^21^ Regarding pharmacological treatment options, Terlipressin (H01BA04) has also been used successfully to treat post-bypass vasoplegia, although it should be noted that this agent is commonly used in Europe, Australia, and New Zealand, but is not approved by the FDA for this use in the United States and Canada.^21,22^ Agonism of arginine vasopressin receptor 1a and subsequent vasoconstriction appears to play the primary role in treating vasoplegia; Terlipressin has more selectivity for the arginine vasopressin receptor 1a than does Vasopressin.^23^ At this moment, there are no randomized controlled trials or large retrospective studies assessing Terlipressin or Vasopressin to specifically treat vasoplegia during Spine Surgery.^9,10^ Nevertheless, according to current cardiothoracic studies, it is acceptable to consider a prophylactic infusion of vasopressin in patients with multiple risk factors for vasoplegia. Moreover, as has been established for the management of neurogenic shock, presence of arterial hypotension in the setting of normal heart rate necessitates intravascular volume loading with crystalloids and colloids within the first 24-48 hours.^11^

Surprisingly, despite the occurrence of vasoplegia and changes in intraoperative neurophysiologic signals, we ultimately found no severe postoperative neurological deficits or major complications in our cohort, aside from one pulmonary embolism. However, it can be surmised that had circulatory compromise gone untreated, severe adverse outcomes could have been expected. A raised awareness and clinical suspicion for the phenomenon of vasoplegia might be beneficial even in surgical specialties outside of cardiothoracic surgery, where this pathogenicity has become well recognized.

Unfortunately, vasoplegia suffers from lack of a unifying clinical definition within much of medical literature. However, if there is unexpected persistent hypotension (MAP <60 mmHg) despite adequate fluid resuscitation (CVP >10 and <15 mmHg with pulse pressure variation in the arterial line <12 mmHg), there should high index of suspicion for this condition. For that reason, any patient undergoing complex spine surgery >6 levels, should have invasive arterial pressure monitoring and central venous access with monitoring of CVP. If feasible, minimally invasive arterial waveform analysis of continuous cardiac output would provide the more reliable guidance for fluid administration and initiation of vasopressors, as well as determination of the cause of hypotension.

Owing to the relative rarity of this complication, analysis of potential risk factors for the development of vasoplegia in the present study was precluded by our relatively small final sample size. Note that any patients with underresuscitation, cardiogenic or obstructive causes or known neurologic (such as spinal cord injury) or systemic confounders (such as sepsis) were intentionally excluded to allow for the clearest possible description of the pathogenicity possible. A more uniform approach to the description of vasoplegia would benefit the present understanding of the underlying pathophysiological mechanisms, risk factors for its development, and establishment of a more formal severity grading scale to help guide pharmacologic intervention, and possibly also prevention. All of our patients were on preoperative antihypertensive medications and possible adverse interactions with intravascular receptors in a complex surgical scenario may play a not yet fully appreciated role in vasoplegia. Another, barrier for recognition has been the slow adoption of more sophisticated intraoperative physiologic monitoring tools that allow for real-time intravascular resistance calculations (ie Pulse index Continuous Cardiac Output monitoring) in specialties outside of Cardiac Surgery, rather than relying upon commonplace tools such as postoperative ECG or echocardiography.

### Limitations

The major limitation of this current study is the small eventual number of patients derived from our very thorough review of a sizeable source cohort under elimination of any patients with possible confounding factors. This resultant small group, however, makes a formal stratified risk analysis for the event of vasoplegia and its predicating circumstances meaningless. Patients with a mild form of vasoplegia may have also been overlooked due to our inclusion criteria. Although we did not have continuous cardiac output monitoring available during our study period, all patients had normal echocardiograms and received adequate fluid resuscitation n the perioperative period, as determined by our selection panel, which included an experienced Cardiothoracic Anesthesiologist (JP). In contrast to Cardiac Surgery, with its routine more elaborate routine intraoperative hemodynamic monitoring, the precise recognition of this occurrence in other fields may have historically led to lack of vasoplegia diagnosis in absence of these resources. Other limitations of this study mainly revolve around the retrospective nature of our study with its inherent risks of selection bias and incomplete records. Fortunately, the extensive nature of these surgeries with thorough preoperative preparations and ensuing formalized records collection associated with these more extensive surgeries allowed for a very detailed and comprehensive data collection with little or no attrition of data.

Despite these challenges, we feel our cohort reflects in greatest detail and specificity possible contributory circumstances leading to the undesirable physiologic complication of vasoplegia and explores first reflections on countermeasures for spine patients undergoing major reconstructive surgery. Hopefully greater awareness of this phenomenon and improved intraoperative monitoring systems that allow for dynamic real time vascular resistance calculations will allow for more timely recognition and intervention of this likely underrecognized condition.

## Conclusion

Although rare, vasoplegia following major thoracolumbar spine surgery should be suspected in patients with intraoperative hypotension refractory to fluid repletion. The pathophysiologic mechanism may be related to manipulation or injury of the sympathetic nervous system, and accordingly medical management should entail escalation of blood pressure support with vasopressors. Despite significant vasopressor requirement, the majority of affected patients in the present cohort did not suffer from long-term neurological deficit or major postoperative complications.
